# The employee’s perception of psychological safety: construct dimensions, scale development and validation

**DOI:** 10.1186/s40359-024-02295-3

**Published:** 2024-12-21

**Authors:** Canghong Wang, Yuwen Ning

**Affiliations:** 1 School of Accounting and Finance, Xi’an Peihua University, No.888, Changning Street, Chang’an District, Xi ’an City, Shaanxi Province China; 2 Teaching and Research Support Center, Air Force Medical University, No.169, Changle West Road, Xi’an, Shaanxi Province 710032 China

**Keywords:** Perception of psychological safety, Team psychological safety, Creative self- efficacy, Organisational performance

## Abstract

Although organisations can effectively resist risks and enhance organisational performance by creating a Perception of Psychological Safety (PPS) climate, there is a lack of PPS scales developed under a multidimensional integrated interaction perspective. Based on a review of relevant theories, this paper clearly defines the concept of PPS and proposes a three-dimensional model of PPS, including organisational- level PPS, team- level PPS, and dyadic- level PPS. In this paper, we develop and validate a scale for measuring PPS in the workplace using 12 startups in a relatively economically developed region in the southeastern coastal region of China as the empirical target. The results of the empirical analyses show that the scale we developed has a good level of reliability and consistency, as well as good content validity, convergent validity, and discriminant validity.

**Supplementary Information:**

The online version contains supplementary material available at 10.1186/s40359-024-02295-3.

## Introduction

Entering the new era, innovation has become a national development strategy, and more and more business organizations are also promoting the capabilities of creative self- efficacy (CSE) of individual employees [1]. For example, encouraging employees to collaborate with colleagues and freely express their new ideas, trying innovative working methods, etc., with a view to promoting the sustainable development of the organization and improving its management processes and practices through these measures [2, 3]. Although employees’ freedom to express their new ideas and trying of innovative working methods may bring benefits to the organization to a certain extent, there are certain risks for individual employees. For example, when individual employees practice free expression of new ideas and innovative management methods in the organization, they often challenge the agreed and conventional work procedures in the organization, and are likely to violate the vested interests of other members of the organization [4, 5]. Furthermore, if individual employees fail to practice these innovative management methods successfully in the organization, it will lead to them lose face or being looked at differently by their colleagues [6], and even being treated as a conversation topic after dinner. Alternatively, if these attempts by individual employees result in certain economic or other losses to the organization, they may also have to bear the consequences arising from them. Therefore, due to the existence of such predictable risks, individual employees usually do not actively propose new perspectives and innovative management methods for the organization. In addition, under the cultural background of China, individual employees tend to have a habitual mindset of “being wise and protecting themselves”. Even when they see certain flaws or decision-making errors in the operation of the organization, they dare not speak up and give advice [7]. This to some extent also suppresses the contribution of individual employees’ experiences or learning to organizational development [8].

Meanwhile, with the accelerated development of globalisation, cross-cultural human resource activities have become increasingly frequent, and the research on cross-cultural adaptation has become more and more in-depth, and the concept of psychological safety has also received widespread attention in recent years, and it has been regarded as a key factor in promoting individual and team effectiveness. Berry’s [9] study shows that cross-cultural adaptation involves both psychological and social adaptation, and that traits such as cultural intelligence, individual stability, openness, and factors such as social networks also have a significant impact on the process of cross-cultural adaptation. Clapp-Smith, Vogelgesang, and Avey’s [10] initiated the exploration of the relationship between cross-cultural adaptation and psychological safety, revealing that psychological safety in cross-cultural environments has an important impact on individuals’ adaptability and overall life satisfaction, which reduces cross-cultural stress and improves individuals’ sense of identification with the new environment. At the same time, individuals who excel at cross-cultural adaptation are more likely to provide a psychologically safe environment in multicultural teams, which promotes mutual learning and cooperation across cultures. Liang and Yang’s [11] study also found that cultural attachment has a significant positive predictive effect on psychological safety. Meanwhile, psychological safety, discrimination perception and cognitive closure mediated the effects of cultural attachment on acculturation stress, and the mediating effect of psychological safety was more significant.

Xu et al. [12] found that individuals whose sense of safety in ontology was threatened preferred to attach to their hometowns which could provide a routine life and construct personal identity, thus seeking to recover their sense of safety in ontology, indicating that an individual’s attachment to their hometowns could alleviate the individual’s sense of safety.The concept of ontology comes from the philosophical field, whose etymological origin (onto) is the Greek word for existence, and is defined as the description of objective existence in the world ^13^, which is people’s perception of their own existence. It is defined as the description of objective existences in the world [13], and is people’s perception of their own existence. The concept of ontology safety proposed by Giddens [14] is used to measure the psychological state of people’s perception of the continuous stability of the environment and the continuous self-identification of themselves, which is derived from the individual’s psychological feelings in the process of his/her own interaction with the environment. On the one hand, the stable interaction between the individual and the environment allows people to establish a sense of trust and stability with the environment, i.e., to believe that the environment in which they live is stable, orderly and predictable; on the other hand, the sense of trust and stability formed between the individual and the environment helps people to form the confidence in the continuity of self-identity, i.e., to believe that there is a continuity between the past, present and future selves, and that they are the individuals who develop stably, thus generating a sense of safety and achieving a state of ontology safety. It can be seen that in the context of globalisation, the combined effect of cross-cultural adaptation, ontological safety and psychological safety is of great significance for organisational effectiveness and personal development.

Numerous studies have fully demonstrated that in a working climate with perception of psychological safety (PPS), individual employees are often able to speak freely without worrying about the consequences. This atmosphere can effectively stimulate individual employees’ self- reflection [15], CSE, and creative problem- solving [16], thereby improving organizational innovative performance [17, 18]. In this psychologically safety work environment, individual employees can freely express their ideas, voluntarily provide the organization with the information they have, dare to express new perspectives, and try innovative management methods and work processes. The work atmosphere of PPS, is particularly important in the healthcare industry. For example, nurses with a high perception of PPS dare to prevent doctors from using medication incorrectly, without worrying about the authority of doctors hindering their career. This can effectively reduce doctors’ medical errors in the diagnosis and treatment process, reduce the reputation and other losses caused by hospital errors, and also effectively enhance patients’ trust in the diagnosis and treatment process and improve doctor- patient relationships [3, 19]. Therefore, it is necessary to understand the benefits that employee psychological safety brings to the organization, as well as the factors that may lead to a decrease in employee psychological safety, which will help organizational leaders design the best work environment to provide beneficial results to their organization to the maximum extent possible. Today, under the impact of the COVID-19 and international trade barriers and other adverse factors, our business organizations in China are studying how PPS can improve employees’ CSE, which is not only of great practical significance to organizational performance and development, but also related to the survival of the organization.

In the Western culture, most scholars study PPS at the team level. They believe that team members can achieve team learning and innovation by freely expressing, venting their dissatisfaction and arguing about different views. However, these behaviors of team members may also lead to interpersonal risk [20]. Therefore, team members are often reluctant to take these actions due to concerns. In order to eliminate their concerns, it is necessary to create a team climate of PPS. At present, most scholars use Edmondson’s 7-item scale for research on Team Psychological Safety (TPS). This scale is developed based on strict scale construction protocols and has undergone extensive empirical testing, always proving its strong content and structural effectiveness. However, research on how various factors at the dyadic, team, and organizational levels interact to affect psychological safety is still scarce. Existing cross level studies are only used to study how team level psychological safety predicts individual level outcomes [21], and how team level psychological safety interacts with individual level variables to predict team outcomes [22]. At present, most studies on the impact of PPS in the workplace are conducted in the context of Western culture, which is often characterized by low-level collectivism, power distance and uncertainty avoidance [23]. In this culture, individual employees are more likely to directly express their new ideas and attempt innovative workflows without a worry of PPS. Therefore, PPS has a certain impact on individual creativity, CSE, innovation performance, and other outcomes. For organizations operating in a cultural background with high-level collectivism, power distance and avoidance of uncertainty, the impact of PPS on individual CSE, creative problem- solving and other work results may be more significant [16]. In this culture, it is not common for individual employees to openly express different opinions or try new ideas, as engaging in such behavior incurs more social costs compared to Western culture. For example, different voices may cause employees to lose their self- esteem, trust, and risk being ostracized by members of other groups [24]. However, this study suggests that there is too much uncertainty in whether PPS has a greater impact on the outcomes of individuals, teams, and organizations operating in different cultural environments, and further exploration is needed. If the Edmondson’ scale is continued to be used in the context of localization in China, it may lead to distortion of research results due to cultural differences. If the scale is continued to be used, it may also hinder progress in this field. Therefore, we must consider integrating various factors of team, organization, and dyadic level in the context of China, and develop a PPS measurement scale in the context to explore the development status of PPS in Chinese organizations, so that organizations can design more suitable organizational environments and management measures for Chinese employees, in order to help employees and teams work more effectively.

In order to enhance our understanding of the mutual influence between different cultures and levels of analysis on PPS, we would explore organizational- level PPS, team- level PPS, dyadic- level PPS, team trust, supportive leadership behavior, supportive organizational practices. We would also review the basic theories and core concepts of psychological safety in relevant literature on relationship networks. At the same time, we also explore the development of psychological safety from aspects such as organizational level, team level, and dyadic level’s integration, interactivity, and systematicity. We also explore how these developments affect the work outcomes of dyadics, teams, and organizations, and identify the important influencing variables of psychological safety and conduct systematic research on the important variants affecting PPS in interaction (organizational- level PPS, team- level PPS, and dyadic- level PPS) and seek empirical evidence to support it. At the same time, we conducted reliable measurement and empirical research on the relevant concepts of PPS. Based on this, our research were conducted from the following aspects: (1) Collecting and organizing existing literature on psychological safety, systematically analyzing and summarizing its core concepts and construction of the concept dimension, and effectively identifying and integrating them. (2) Summarizing various important factors that affect psychological safety, and effectively distinguishing and systematically integrating these important factors to form a recognizable theoretical framework. (3) This study combined interview and questionnaire methods to develop a PPS measurement scale under the cultural background of China. Exploratory Factor Analysis (EFA) and Confirmatory Factor Analysis (CFA) were used for reliability and validity testing, forming a measurement model for PPS. (4) We have verified the impact of PPS on individual CSE, and discussed the future application and inspiration of this scale.

## Basic theory: the connotation and expansion of PPS

Schein and Bennis [25] were the earliest scholars to propose PPS in the workplace, and their research first found a positive impact relationship between individual PPS and organizational innovation performance. At present, research on PPS in the academic community is mainly conducted from three dimensions (individual dimension, team dimension, and organizational dimension). Professor Edmondson from Harvard University in the United States first proposed TPS. In an article published in the *Quarterly Journal of Management Science* in 1999, she clearly defined the concept of TPS. She believed that team members often point out the mistakes of other members, express dissatisfaction, and argue about a certain viewpoint during the learning or work process. These behaviors can promote team learning and achieve team innovation performance. Of course, Edmondson’s research on PPS was conducted at the team level, and she defined TPS as “a perception of whether it is safe to engage in interpersonal adventure behavior within a team”. It can be seen that Edmondson’s research is a type of team traits. Later, Tynan [26], Detert and Burris [4] redefined the concept of TPS based on Edmondson [2] and jointly proposed self psychological safety and others’ psychological safety, which is the classic Dyadic Psychological Safety. Self psychological safety is an individual’s perceived self-awareness, such as whether they perceive others to blame, respect, and trust them [4, 26]. Therefore, individuals can judge whether to engage in interpersonal adventure behavior with others based on their self-awareness. The others’ psychological safety is that when individuals perceive that they may pose a threat to themselves, they will adopt a distant or evasive approach to avoid normal interpersonal interactions with others [4, 26]. Conversely, it will manifest as a positive tendency towards interpersonal interaction. In short, the PPS of others refers to the positive or negative interpersonal interaction tendencies that individuals adopt based on their own perception to determine whether it is safe to interact with others. At this point, Tynan [26] made Edmondson’s construct of PPS concrete at the level of work interaction. In this way, the object of PPS is no longer the team, but rather the interaction parties (such as managers and employees), because he advocated that the strongest variable that affects psychological safety is leadership behavior. In other words, Tynan’s research extended Edmondson’s definition of psychological safety. The difference lies in the fact that the introduction of Kahn’s [27] concept of individual PPS by Tynan [26], which mainly focuses on the PPS of individuals, colleagues, and managers, as well as the PPS of others. When an individual presents themselves, they consider whether their appearance, social status, and career will be negatively affected by others. Edmondson [2] mainly focuses on the relationship between individuals and teams, that is, the perception of TPS. At present, although there is still significant controversy over the definition of TPS, most research in this field uses or references Edmondson’s [2] scale. Edmondson’s [2] research emphasizes the connotation of TPS and is also a prediction of team members’ subjective willingness to take risks, rather than making them feel comfortable. In addition, Brow and Leigh [28] formally introduced PPS at the organizational level for the first time. They defined PPS as one of the influencing factors of organizational atmosphere, defined as the degree to which employees psychologically perceive their environment as safe. There are also other studies that extend PPS to the organizational level. For example, Carmeli and Gittell [29], Carmeli et al. [30] define organizational PPS as an organizational norm and procedure that guides and supports individuals within an organization to be more open and trusting, and engage in more meaningful interactions.

At the work environment/team level, a large amount of research on PPS has begun to explore the connotation of individual PPS. For example, employees in organizational workplaces are more likely to feel psychologically safe when they have mutual trust and supportive interpersonal relationships with colleagues. Later, the PPS of individuals in the work environment was defined as the shared belief held by team members. Subsequently, a large number of studies defining people’s perception of PPS from a team perspective emerged, and most of them have achieved good results. Of course, there are also studies that define people’s perception of PPS from an individual perspective, for example, Kahn [27] developed a research scale on people’s perception of PPS from an individual perspective. The scale has achieved good results through empirical testing. The seven item scale developed and verified by Edmondson [2] is one of the most popular scales. She used the qualitative research method to find the common view among team members: that is, the common view of team members is that they will not refuse to help other members because they only care about their own work, but they will care about each other like social members, have a positive willingness to help each other, and respect each other’s ability. Indeed, research on psychological safety at the team level has described team traits rather than team member traits. There is also no effective distinction between safety and trust in defining the boundaries of TPS.

In summary, we can strengthen our understanding of the atmosphere of PPS from three aspects: (1) Individual employees believe that expressing opinions in the workplace is safe, and they perceive that colleagues in the team welcome and expect their opinions; (2) In the workplace, individual employees do not need to focus on self-protection and can focus on valuable discussions without worrying; (3) In the workplace, even if an individual employee has some flaws in his free expression of an event, he does not need to worry about being despised or causing difficulties to his career. In short, the sense of safety in the workplace is not about keeping team members in a comfortable area, but about allowing them to enter the learning area and actively engage in mutual learning and collaboration.

## Construction of the concept dimension of the PPS based on qualitative mete-analysis

Given that most of the current research on PPS is presented in the form of empirical studies, this paper draws on the idea of “integration” in the qualitative meta-analysis method to refine and summarise the published empirical studies in order to construct a more universal conceptual framework of PPS. Due to the different purposes, perspectives and analytical strategies of the existing empirical studies, the existing results are rather trivial and isolated. The qualitative meta-analysis method emphasises the unification of empirical studies with similar themes, re-analysing, comparing and interpreting findings and evidence from existing studies, and consolidating the categories that have emerged in different studies to enhance dialogue and accumulation of knowledge. qualitative meta-analysis can also identify previously undiscovered implicit phenomena from empirical research and provide new nutrients to the current system of knowledge. The qualitative meta-analysis method is therefore particularly well suited to the current ‘fragmented’ theoretical development of PPS research and contributes to the development of an integrated conceptual framework for PPS.

### Selection of the qualitative mete-analysis samples

The source of data for the qualitative meta-analysis method is existing empirical research findings. To ensure data quality, the sample was limited to published empirical research papers, while articles published in popular publications, consultancy reports or news reports were excluded. The search was conducted in English and Chinese databases such as CNKI and Web of Science, and the search terms “psychological safety”, “PPS”, " individual psychological safety”, “organizational- level PPS”, " supportive organizational practice”, “team traits”, “individual traits”. “team-level PPS”, “dyadic-level PPS”, “team trust’, and “relationship network”, etc. The search terms in the English literature are standard translated versions of the Chinese terms. The year 1996, when Brown and Leigh [28] published in The Journal of applied psychology, was used as the starting year, and the search period was set at 1996–2022. The initial search yielded 89 empirical studies that met the requirements. Further, drawing on Rousseau et al.‘s [31] recommendations for exclusion rules for qualitative meta-analysis studies, the following types of literature were excluded: (1) studies published in basic and applied psychology within the discipline of psychology, such as those in the areas of personality, behavioural habits, artificial intelligence, IQ, personality, etc.; (2) studies published in Research in medical disciplines on subjects related to neuroscience, medicine, philosophy, biology, religion and other disciplines that explore how physiological or psychological effects can affect an individual’s mind; (3) published research that occurs in many areas of everyday life - family, education, health, society, etc. This resulted in the exclusion of 68 pieces of literature. Finally, after eliminating some of the less relevant studies, a total of 19 academic literature on PPS was obtained, containing 19 relevant empirical research articles.

### Analysis of the qualitative mete-analysis data

The operational steps of the qualitative meta-analysis were followed to execute the analysis process of extracting, coding and categorising the empirical study sample. Firstly, the original statements and key findings related to psychological safety were identified in the included empirical studies, resulting in 122 extracts of relevant words and phrases. These statements were then coded, following the recommendations of Gioia [32] and others for qualitative data analysis, using a two-stage coding approach. The first stage of coding was carried out by ‘labelling’ the statements to fit rigorously into the original data of the empirical study and to give a realistic picture of the observed phenomena. Each segment was labelled individually while retaining as much of the original meaning of the statement as possible, and the original labels were repeatedly identified and compared. In turn, some labels that were less relevant to psychological safety were eliminated, and semantic repetitions were merged to initially refine a more generalised initial category. This phase of the coding process was conducted independently by both researchers and was controlled and explored at the end of each empirical study coding. A total of 108 valid tags and 46 initial categories were generated at this stage.

The second stage is the process of categorisation, i.e. the generalisation of initial categories with juxtapositions and affiliations into more abstract higher order dimensions. This is a process of theorising, in which full reference is made to constructs and theories from the existing literature. For example, drawing on the concept of ‘organisational-level PPS’ in the field of psychological safety, initial categories such as ‘organisational support’ and ‘supportive organisational practices’ are included in “The ‘organisational level of PPS’ emphasises the idea that ‘supportive organisational practices promote psychological safety through social learning’ [28], and is therefore distilled into the term ‘organisational- level PPS’. higher-order dimension of ‘PPS at the organisational level’. In addition, the coding process focused on phenomena that had not been adequately summarised in the existing literature, and explored their hidden meanings. After repeated comparisons and iterations, the three themes of organisational-level PPS, team-level PPS and duality-level PPS were obtained (see Table 1), which can explain the conceptual content of PPS in a clear and complete way.

**Table 1:** The main research perspectives, main researchers, and research conclusions on PPS

Dimension	Research perspective	Main researchers	Conclusion
Organizational-level PPS	Organizational PPS.	Brown and Leigh [28]; Carmeli et al. [30]; Carmeli and Gittell [29].	Supportive organizational practices promote PPS through social learning processes.
	supportive organizational practices.	Carmeli and Zisu [33]; Chen et al. [34]; Singh et al. [35].	
Team-level PPS	TPS and team trust.	Edmondson [2, 36].	Supportive leadership behavior and positive leadership behavior can effectively promote PPS.
	Supportive leadership behavior.	Edmondson [2]; Hirak et al. [37]; Carmeli et al. [18]; Detert and Burris [4].	
Dyadic-level PPS	Relationship network.	Carmeli and Gittell [29]; Carmeli et al. [30]; Zaman and Abbasi [21]; Ozer and Zhang [38]; Brueller and Carmeli [39]; Burris et al. [40]; Carmeli [41].	Individuals have a strong network of relationships and the inherent social resources within these networks that effectively promote their individual PPS.
	Individual PPS	Kahn [27]; Tynan [26]; Detert and Burris [4]; Carmeli et al. [30].	

### Interpretation of the qualitative mete-analysis results

In the following, each of the three dimensions of PPS is described, and the logical relationships between the dimensions are explained to form a complete construction of the conceptual system of PPS.

At the organizational level, PPS is considered to be an employee’s perception of the organizational climate [28]. This perception includes supportive organizational practices perceived by individuals, such as clearly positioned work roles and freely expressed organizational climate. In other words, when an organization has more supportive management practices, it gives its employees clearer work goals, and employees are willing to express their opinions freely. Therefore, individuals will perceive a higher level of PPS from the organization. In addition, Brown and Leigh [28] and other scholars [29, 30] have aggregated Edmondson’s TPS at the organizational level as a tool for studying organizational PPS, which is also an effective extension and extension of the TPS- scale. It should be clarified that both Brown and Leigh [28] and Edmondson [2] tend to reflect the contextual traits perceived by employees, conveying a shared belief, but their definitions of the objects of PPS are different. However, the research based on Edmondson’team level measurement scales has replaced teams with organizations. They have all measured PPS within the organization, essentially simply summarizing the measured scores at the organizational level based on the high intra-class correlation coefficients (ICC) between individual members of the organization. Although these studies suggest that individuals’ PPS can be aggregated at the organizational level, it is questionable whether this exists in all organizations. We believe that an organizational atmosphere with high ICC and PPS among individual members may exist, but we must also consider that employees’ PPS is also influenced by leadership ability and team traits. Therefore, it is unlikely that high ICC agreements can be reached among organizational members. The objective fact is that in larger companies, employees are generally less willing to share leadership experience and team norms. Of course, we cannot rule out the possibility of this atmosphere of PPS may exist in smaller organizations in which members of the organization need to collaborate regularly to complete specific work tasks. Therefore, further empirical testing is needed to determine whether this PPS exists in organizations with high ICC protocols or strong organizational culture. There is increasing evidence at the individual level that supportive organizational practices are positively correlated with employees’ work outcomes (organizational commitment and work performance), as organizational commitment and work performance enhance employees’ PPS [42–44]. Employees’ perceived organizational support, guidance, and diverse practices promote work outcomes through mediation mechanisms of PPS [33–35]. In addition, supportive organizational practices promote PPS through social learning processes [33]. The social identity theory suggests that the implementation of workplace diversity practices promotes PPS through employee and organizational identification [35].

At the team level, in the study of PPS, we need to integrate PPS and team trust into one phase for description. Edmondson [2] and his collaborators first conceptually distinguished the similarities and differences between TPS and team trust. They believed that TPS includes trust, but at the same time goes beyond trust. They distinguished three common aspects of PPS and trust in terms of concept: (1) both TPS and trust describe the internal psychological state of individuals who perceive risk or are prone to blame; (2) Both TPS and trust require making decisions that minimize negative impacts; (3) TPS and trust have potential for positive results of group fitting organization. At the same time, Edmondson and his collaborators distinguished trust from three aspects of TPS: time frame, object of focus, and level of analysis [36, 45]. Specifically, in terms of time frame, team’s PPS is mainly concerned with immediate criticize and disdain in person without considering without considering the consequences of avoidance and interpersonal risk. Trust, on the other hand, takes into account the consequences of different time periods over a longer time span, and may even consider future consequences. In terms of target audience, TPS is set to ensure that others do not have doubts about themselves. In interpersonal risk situations, the direction of action is from others to oneself, while trust is set to ensure that the target audience does not have doubts about others. In interpersonal risk situations, the direction of action is from oneself to others, with a focus on the potential behavior of others. At the analytical level, the team level PPS is at ternary level while trust is at the dyadic level, so there is conceptual inconsistency. In addition to directly using the Edmondson’s [2] scale or adapting its version, some researchers have also developed their own scales on TPS, such as the empirical research by Hirak et al. [37]. The difference is that Hirak et al. [37] used data from Anderson and West’s [46] measurement of psychological strength climate in a unit. These studies set a high ICC among team members, and their cognition of their internal PPS is summarized at the team level for research. For example, research on supportive leadership behaviors mostly focuses on the individual and team levels, and has also confirmed the impact mechanism of supportive leadership behaviors on work outcomes through PPS. Research on supportive leadership behavior has found that leaders’ individual traits (such as inclusiveness, support, credibility, openness, and behavioral integrity) has a strong impact on employees’ PPS [47, 48]. At the team level, employees’ collective perceptions supported and guided by team leaders, trust in the leader, leader’s Inclusiveness, and behavioral integrity of the leader, all of which can promote team level outcomes such as team learning behavior, team performance, and quality improvement work, and reduce errors among team members by developing a perception of PPS [2, 19, 37, 49]. Other studies have also found that positive leadership styles (transformational leadership, ethical leadership, change-oriented leadership, and shared leadership) are positively correlated with employee voice behavior, team learning, and personal learning through the mediating mechanism of PPS [21, 22, 50]. Leaders who value participation, personnel, and production can use binary discovery methods to cultivate a high level of PPS [51, 52], rather than using group discovery methods and an improvement oriented management style [53]. This explains why there may be a significant correlation between supportive leadership behavior and PPS, as previous studies typically rely on key principles in social learning theory [54]. Based on this theory, researchers believe that by listening, promoting support, and providing clear and consistent guidance to subordinates, leaders can demonstrate to subordinates that taking risks and engaging in honest communication are safe [37, 49]. We believe that the social exchange process may increase PPS, but we believe that these effects are likely to be stronger and more lasting, because PPS is established by learning and imitating leaders’ behaviors, rather than displaying them at a certain point in time in exchange for specific leadership behaviors.

At the dyadic level, Kahn [27] believes that PPS is an individual’s perceived self-awareness and psychological state, which affects their internal psychological motivation, which in turn relates to the individual’s degree of dedication. Therefore, Kahn [27] believes that when employees experience a higher level of PPS, they will have a higher level of work engagement. In other studies, most of them only measure the dyadic correlation between individual employees and the organization/team, which is about the individual perspective of PPS in the team/organization. For example, Detert and Burris [4] constructed a study on PPS with supervisors and employees as the focus. He used Edmondson’s scale to adapt teams into organizations to examine individual employees’ PPS within the organization [30]. Some researchers have also developed their own measurement scales, such as Singh et al. [35], Detert and Burris [4], etc. The continuously increasing research at individual, team, and organizational levels has identified social support and the inherent social capital (resources) in relationship networks as key determinants of PPS. At the dyadic level, research has found that the mediating mechanism of PPS affects individuals’ learning and participation [29, 30]. Similarly, relationship networks and the inherent social support and resources within these networks promote PPS and contribute to team learning, performance, and innovation. For example, researchers have found that the key driving forces behind PPS and its outcomes are: prior interaction levels, familiarity among team members, quality of social relationships measured through trust, network ties and collective thinking, high-quality relationships between team members and external members, identity of core members [38–40, 52, 55]. At the organizational level, research has found that the strength of social networks among organizational members is positively correlated with their learning ability in the failure of PPS development [41]. Although most studies in this field do not clearly explain why strong relationship networks and the inherent social resources in these networks promote PPS, a few researchers have proposed that social relationships may generate PPS through social learning processes [41].

In a word, scholars at home and abroad have extended the research on PPS to many factors (organization, individual, team, team trust, supportive leadership behavior, supportive organizational practices, and relationship networks). This study analyzes the integration, interactivity, and systematicity of PPS at the organizational level (including organizational and supportive organizational practices), team level PPS (including team, team trust, and supportive leadership behavior), and dyadic-level PPS (individual and relationship networks), and summarizes the research on PPS by domestic and foreign scholars. In Table 1, we summarize the main research perspectives, main researchers, and research conclusions on PPS.

## Scale development

### Research technique: initial scale generation

The focus of this paper is on the development and validity testing of the PPS measurement model. We followed Churchill, Gilbert, and Iacobuoci’s [56] scale development procedure, developed a measurement indicator pool based on a literature review and expert panel interviews, and refined the relevant indicators on the basis of the content validity of the PPS, progressively eliminating the unqualified indicators.

#### The choice of research background

Although the PPS defined and the key dimensions identified in this paper are applicable to all types of PPS, it is not easy to find a universally applicable set of measurements in practice because different types of PPS may vary significantly. In this study, we chose startups and their employees with a strong willingness to predict their organisational climate both subjectively and objectively to satisfy the initial scale’s measurement requirement of PPS as the context of the study. This choice was made because of the growing importance of employee PPS in the innovative performance of firms. Specifically, we developed and validated the PPS scale using PPS in organisational, team and dyadic contextual features as examples.

#### Preliminary research

As a preliminary study, we organised group interviews with two comparison groups. One group included five national experts in the field of psychology. The other group consisted of five management specialists, such as human resource management, with more than 15 years of managerial experience in organisations. These interviewees were selected to be as representative as possible of the diverse characteristics of the PPS, such as different organisations, age, income, educational background and experience. The main objectives of the interviews were (1) to ensure the appropriateness of the measurement model, (2) to ascertain that the sample was selected to reflect changes in PPS, and (3) to generate specific measurements for each PPS dimension.

#### The generation of indicators

Based on a systematic review of previous relevant literature, a review of existing measurement models, and group interviews, and through the use of an inductive approach [57], we derived as many initial indicator libraries as possible for measuring PPS, identifying a total of 36 indicators for measuring PPS, with each dimension including 12 indicators. In the group interviews with the comparison group, as described above, the researcher first described to the respondents the basic meaning of PPS at the organisational, team and dichotomous levels and gave examples, and then asked each of them to list the most appropriate metrics describing each of the dimensions. In the initial pool of metrics, 16 metrics were mentioned frequently. Further references were made to We further refer to Edmondson [2], Detert and Burris [4], Tynan [26], Kahn [27], Brown and Leigh [28], Carmeli and Gittell [29], and Hirak et al. [37] and documented these indicators for content validity testing.

#### Content validity

In this study, we invited five researchers (including two senior researchers, one senior human resource manager, and two professors of psychology) to cooperate with us in testing content validity. Based on informing these experts about the concept of PPS and its dimensions, they were asked to rate them according to how well each indicator describes the fit of the corresponding dimension: 1 means very representative, 2 means representative to some extent, and 3 means not representative at all [58]. Finally, only indicators that were considered representative at least to some degree were retained. Through testing, the number of indicators was finally determined to be 16, which is very much in line with the collated results from the group interviews described above.

#### Preliminary research

Firstly, a preliminary questionnaire was distributed among a number of selected firms prior to the formal large-scale survey, and the questionnaire was modified through their feedback. A Likert 5-point scale (from 1 to 5 representing from totally disagree to totally agree respectively) was used for all variable indicators. In the exploratory research, 50 questionnaires were distributed to the employees of the selected companies and 46 were returned (92 per cent return rate). Of these, 10 questionnaires were considered invalid due to incomplete data and a total of 36 questionnaires were used for preliminary data analysis. After refining the measurement process according to the theory of Churchill et al. [56], there was good evidence that: the content validity criteria were all well met. Of these, one indicator was excluded and the remaining 15 variable indicators were entered into a formal large-scale survey and data analysis. Of these, five were organisational level PPS, four were team level PPS and six were dyadic level PPS (see Appendix 1).

### Selection of measurement objects and measurement methods

We collected data of the initial scale by manually distributing questionnaires offline. Due to the fact that the measured object of this scale is organizational environment or organizational atmosphere, it is necessary to consider that the measured enterprises have a high level of inclusiveness and openness. In this study 12 entrepreneurial enterprises from relatively developed coastal areas in southeastern China for questionnaire distribution were selected. A Likert 5-point scale (from 1 to 5 representing from totally disagree to totally agree respectively) was used for all variable indicators. These selected startups come from Guangzhou (4), Shanghai (4), and Suzhou (4), respectively. These selected entrepreneurial enterprises have a strong willingness to predict their organizational atmosphere both subjectively and objectively, which meets the measured requirements of the initial scale.

The time interval for this questionnaire survey is from mid June 2020 to the end of October 2022. We distributed and collected questionnaires in a face-to-face manner. Under the guidance of the general manager of the company, the question staff anonymously answered the questionnaires during regular meeting breaks. After the meeting, department heads collected the questionnaires, and the company manager assisted the general manager in summarizing and submitting them to us. A total of 858 questionnaires were distributed in this survey, with 842 valid questionnaires remaining after excluding invalid ones. The recovery rate for valid questionnaires was 98.14%. The demographic information of the questionnaire used in this survey is as follows: 586 males (69.60%) and 256 females (30.40%); The average age is 31.55 years old; 12 people with high school education (0.14%); 31 people have a college diploma (8.08%), 620 people have a bachelor’s degree (73.63%), and 179 people have a graduate degree or above (21.26%); From the positions of the respondents within the organization, there are 557 general staff members (66.15%), 36 supervisors (4.28%), and 24 managers (2.85%). There are 12 general managers or chairpersons (1.43%). All demographic information indicates that it is in line with the actual situation of the tested enterprise and has strong representativeness.

### Transparency, openness, and method

We describe our survey plan, all data exclusions (if any), all manipulations and all measures in the study, and followed the methodological checklist of the BMC Psychology. All data, analysis codes, and study materials are available from the corresponding author on reasonable request. Data analyzed using SPSS 22 for item analysis, factor analysis, reliability analysis, and a test of the predictive validity of the scale used regression analysis. The design and analysis of this study were not pre-registered.

## Analysis of the scale

### Item analysis

Through a quantitative analysis of the 15 items of the initial scale, it was found that each item had good social identity. Each item has a relatively stable mean value range (3.722 to 4.386), indicating that there is no outlier for each item. The accuracy range of each question item (0.511 to 0.723) is evenly distributed, and no outlier was found. The distribution range of comprehensibility levels for each question item is 0.713 to 0.824, indicating that each item has a moderate level of comprehensibility, being easy to understand, and no ambiguous items were found.

### Exploratory factor analysis

The total sample size of this study is *n* = 842. In order to perform EFA and confirmatory factor analysis (CFA) using different data, we randomly divided the total sample data into two equal sets of data, with each sample size *n* = 421. Firstly, we conducted EFA on the measurement items of the initial scale using one set of data. The results showed that the KMO of the scale was 0.906 (higher than the standard value of 0.700), indicating that the sample size of the questionnaire was sufficient; the significance level of its Barrett spherical test value is 0.000 (less than 0.001), indicating that it is suitable for CFA; x^2^ = 3212.322, df = 371. When using SPSS 22 for EFA, principal component analysis and maximum variance oblique rotation method were used. During this process, a total of three factors with feature roots greater than 1 were extracted, and these three feature root factors accounted for 58.332% of the total variance. In EFA, we removed items with factor load values less than 0.500 [56–59], or items with multiple loads and different factors. A total of 1 inappropriate items were removed, the deleted item was “in the workplace, we are free to express our opinions when we communicate about specific work tasks.“, leaving 14 items. Subsequently, after repeated EFA, we did not find any problematic items. In the end, the remaining 14 items of the PPS-Scale were distributed among 3 factors, explaining a total variance of 62.466% (see Table 2). The three factor model is consistent with the hypothesis concept corresponding to the questionnaire measurement, that is, the PPS-Scale is composed of three factors: organizational level PPS, team level PPS, and dyadic level PPS. The variance contribution rates of these three factors are 18.543%, 21.487%, and 22.436%, respectively. The specific items finally determined are shown in Table 2. F1 (organizational level PPS) dimension contains 4 items, F2 (team level PPS) dimension contains 4 items, and F3 (dyadic-level PPS) has 6 items.

**Table 2:** Factor matrix of PPS-Scale (*n* = 421)

Item			Factor loading	Item			Factor loading
PPS at organizational level (F1)				PPS at team level (F2)			
POPS1	In the workplace, we fully understand our work goals or roles.		0.713	PTPS1	In the workplace, our team members become familiar with each other through social discussions.		0.633
POPS2	In the workplace, we can freely express our views without taking risks.		0.726	PTPS2	In the workplace, our team leaders always provide me with clear and consistent work guidance plans.		0.698
POPS3	In the workplace, we always receives organizational support and assistance when we encounter difficulties.		0.706	PTPS3	In the workplace, our team leaders always refer to the opinions of their members when making decisions.		0.701
POPS4	In the workplace, we can use different methods to solve work problems		0.721	PTPS4	In the workplace, our team leaders always share the joy of success or the frustration of failure with us.		0.796
PPS at dyadic level (F3)							
PIPS1	In the workplace, I can be my true self.		0.791	PIPS4	In the workplace, the work I do has a significant impact on my team’s performance.		0.692
PIPS2	In the workplace, my team cares about my health.		0.782	PIPS5	In the workplace, I can choose the work style that suits me.		0.705
PIPS3	In the workplace, my manager considers my perspective when making decisions.		0.699	PIPS6	In the workplace, I can always perceive a clear development of the company.		0.598
Factor naming		PPS at organizational level (F1)			PPS at team level (F2)	PPS at dyadic level (F3)	
Eigenvalue		3.336			3.586	3.411	
Variance contribution rate		18.543%			21.487%	22.436%	
Cumulative variance contribution rate		18.543%			40.030%	62.466%	

### Confirmatory factor analysis

When conducting CFA, the results see Table 3, we used another set of sample data (*n* = 421) to test the fit between the proposed model and the observed model. At the same time, we proposed two comparative and conceptual models to better validate the accuracy of our research model. Firstly, M1 represents a single factorial model, which assumes that all 14 items represent a sense of PPS in one dimension. Correspondingly, M2 represents a two-factor model, while M3 is a three-factor model. The results of EFA, which are constructed based on the theory of PPS, are three factors: PPS at the organizational level, PPS at the team level, and PPS at dyadic level. CFA was conducted on the three factor model of PPS, and the analysis results showed that M3 had x^2^ = 496.113, df = 168, x^2^/df = 2.953 (the value of x^2^/df was less than the standard value of 3), indicating that M3 was optimal; RMSEA = 0.069 and SRMR = 0.041 (both less than the standard value of 0.800 [56–59]; CFI = 0.916, TLI = 0.923, both CFI and TLI values are higher than the standard value of 0.900 [56–60]. According to the comparison analysis of the observation data and fitting index of each model in Table 3, it was found that compared with other models (M1, M2), There is a significant increase in both TLI and CFI values in M3, with a significant decrease in x^2^/df values, as well as a significant decrease in RMSEA and SRMR values. In summary, it can be seen that M3 is superior to the comparative model, and the factor model analysis results of the perception of psychological safety Scale tend to support the three factor model M3 with good fit [56, 57]. Therefore, the structure of the conceptual model (M3) has been effectively validated.

**Table 3:** Fitting degree of the PPS-Scale (*n* = 421)

Model	x^2^	df	x^2^/df	RMSEA	SRMR	CFI	TLI
Three-factor model (M3: F1, F2 and F3 as one factor, respectively)	496.113	168	2.953	0.069	0.041	0.916	0.923
Two-factor model (M2: F1 and F2 combined as one factor, and F3 as one factor)	630.585	173	3.645	0.093	0.046	0.891	0.843
One-factor model (M1: F1, F2, and F3 combined as one factor)	864.012	178	4.854	0.115	0.505	0.823	0.802

### Analysis of reliability and validity

When conducting reliability analysis, we used sample data (*n* = 421) from CFA to analyze the reliability of each factor in the PPS-Scale. We found that Cronbach’s α = 0.801 (see Table 4) which indicates that the scale has good reliability and measurement quality [61]. Simultaneously we also found that all Cronbach’s α in the PPS-Scale in all dimensions was greater than 0.700 (much higher than the standard value of 0.700) (see Table 4), indicating that the reliability of all dimensions of the total table is good [62], Cronbach’s α of PPS at team level and individual PPS dimensions are all close to 0.800, indicating a very high level of reliability.

**Table 4:** Internal consistency coefficient of factors in PPS-Scale (*n* = 421)

The whole scale	The number of item	Cronbach’s α	The name of factor	The number of factor	Cronbach’s α
PPS-Scale	14	0.801	Organizational-level PPS	4	0.766
			Team-level PPS	4	0.813
			Dyadic-level PPS	6	0.825

When conducting validity analysis, we considered content validity, aggregate validity, and discriminant validity, respectively. When analyzing content validity, this study removed factors with factor load values less than 0.500 or items with overlapping factor loads from EFA. Due to the high retention criteria set in this study, multiple rounds of deletion and optimization were conducted on the scale items during robust CFA. At the same time, the development of this scale followed the standard theoretical paradigm, reviewed sufficient literature, and conducted interviews and research with qualified industry experts and scholars. At the same time, the initial scale items were repeatedly considered, discussed, and optimized to ensure that the PPS-Scale had high content validity. When analyzing the aggregation validity, the AVE range of each dimension in the total table is 0.736 to 0.787 (see Table 5, all exceeding the standard of 0.500). Due to the standard factor loading of EFA and CFA exceeding 0.500 for each dimension of the scale, it indicates that the PPS-Scale has good aggregated validity [56, 59]. When analyzing the discriminant validity, the root mean square of AVE in each dimension of the PPS-Scale was compared with the correlation coefficients between each dimension. The range of values for the root mean square of AVE was between 0.832 and 0.914 (see Table 5), which were greater than the correlation coefficients of its row and column. This indicates that the discriminant validity of this scale is good [56].

**Table 5:** Correlation matrix and validity test for each dimension of the PPS-Scale (*n* = 421)

Structure dimension	F1	F2	F3
Organizational-level PPS (F1)	(0.832)		
Team-level PPS (F2)	0.744	(0.871)	
Dyadic-level PPS (F3)	0.688	0.726	(0.914)
AVE	0.736	0.756	0.787
CR	0.882	0.906	0.934

Note The value in parentheses is the square root of the average variable extraction amount for this latent variable

## A test of the predictive validity of the PPS- scale

An important aim of concept development is to be able to explain or predict phenomena. The purpose of this part of the study is to empirically test whether the PPS- Scale can effectively predict the effect of employee creativity. Through a review of existing research, this study distils the correlates of creativity self-efficacy (CSE) [63]. Therefore, this study preliminarily examined the most fundamental research topic in the theoretical framework of psychological safety, which is whether implementing a work climate of PPS in enterprises will promote the improvement of individual employees’ capabilities of CSE.

### Theoretical background and research hypotheses

Based on the concept framework of PPS in theoretical foundations, the organization’s focus on work climate of PPS will be beneficial for improving individual employees’ professional commitment level and innovative performance. In a psychologically safe workplace, employees can fully utilize their talents and focus on solving practical problems rather than interpersonal relationships, in order to calmly respond to unknown challenges and risks. Research has found that individuals with high self beliefs or expectations continuously stimulate and generate a perception of PPS, which in turn encourages employees to find creative paths to solve difficult problems. In fact, if in a workplace with a perception of PPS, employees will continuously question and reflect on difficulties and problems in their work, and promote the experience and results of reflection, in order to discover the best path to solve thorny problems. On the contrary, if there is no or poor PPS climate in the workplace, employees will lose the motivation for self reflection or reflection, because this lack of self reflection belief can cause employees to become timid and complacent, thereby inhibiting their individual CSE [63]. Therefore, our basic research topic is summarized as: will the implementation of PPS practices by organizations promote the individual CSE of employees? Specifically, it refers to whether the focus on PPS at the organizational, team, and dyadic levels will have a positive impact on the CSE of individual employee in the organization/team. We attempted to preliminarily verify the above issues using the developed PPS-Scale. Therefore, we propose the hypothesis:

#### Hypothesis

*the three dimensions of PPS (organizational*,* team*,* and dyadic levels) can have a positive impact on the improvement of individual employees’ CSE*.

### Research methods

The data for this study were all from 12 start-up enterprises in relatively developed areas along the southeast coast of China. The questionnaires were distributed by the general manager of the questioned enterprises during regular meetings, and collected by department heads. A total of 842 valid samples were analyzed. The questioned subjects include employees at all levels, from ordinary employees to general managers, and have a wide representation. After EFA and CFA, the reliability and validity of the dimensions and items of the scale were satisfying. We used Tierney and Farmer’s [64] 4-item scale as the the Creative Self efficacy Scale (e.g., I am confident in my problem-solving abilities through the application of creativity). The measurement was in the form of 5-point Likert scale, being completely inconsistent = 1 ponit, completely consistent = 5 points, and at the same time, the demographic information (age, gender, financial level and number of years of employment) of the questioned employees was also included as control variables in the analysis.

### Research results

In this study SPSS 22 was used to perform data analysis. The results of CFA showed a good fit. The PPS-Scale’s Cronbach’s α = 0.801, and the Self-efficacy’ s Cronbach’s α = 0.932, both of which indicate that the measurement scales for each variable have good reliability, and the Table 6 shows the descriptive statistics i.e. the results of analysis of each variable.

**Table 6:** Descriptive analysis of variables

Variable	M	S	D	1	2	3	4	5	6
1.Gender	1.553	0.613	1						
2.Age	31.550	4.233	−0.147**	1					
3.Financial level	0.644	0.347	0.046	0.061	1				
4.Years of employment	4.122	0.335	0.005	0.060	0.073**	1			
5.Team-level PPS	4.011	0.224	0.002	0.058	0.071**	0.565**	1		
6.Organizational-level PPS	4.335	0.588	0.013	0.065*	0.070**	0.672**	0.733**	1	
7.Dyadic-level PPS	4.003	0.421	0.016	0.061*	0.069**	0.635**	0.673**	0.693**	1
8.CSE	4.232	0.553	0.162**	0.031	0.230**	0.233**	0.311**	0.273**	0.298**

Note β = Standardized Beta coefficient; ** *p* < 0.010;* *p* < 0.050

According to the regression analysis model results shown in Table 7, the three dimensions of PPS are: organizational level PPS (β = 0.348, *p* < 0.010). PPS at the team level (β = 0.301, *p* < 0.010), PPS at the dyadic level (β = 0.311, *p* < 0.010). Based on the statistical results in Table 7, all dimensions of the PPS-Scale have a significant positive impact on employee’s CSE. The F-values of each regression model reach a significant level, and the regression coefficients of each variable in the model are all positive and significant for the dependent variable. Therefore, the research hypothesis has been validated on all dimensions of the PPS-Scale.

**Table 7:** Regression model of the influence of various dimensions of PPS on CSE

Variable	CSE		
	model 1	model 2	model 3
1.Gender	−0.152**	−0.154**	−0.146**
2.Age	−0.013	−0.011	−0.008
3.Financial level			
4.Years of employment			
5. Team-level PPS	0.301**		
6.Organizational-level PPS		0.348**	
7.Dyadic-level PPS			0.311**
R^2^	0.092	0.106	0.131
adjusted R^2^	0.083	0.114	0.092
ΔR^2^	0.092	0.014	0.013
F	26.304**	32.335**	25.883**

Note β = Standardized Beta coefficient; ** *p* < 0.010;* *p* < 0.050

## Conclusions and inspirations

### Conclusion

In this paper, based on a comprehensive analysis of relevant literature and practical research, we provide a comprehensive definition of PPS and further propose a three-dimensional model of PPS: organisational- level PPS, team- level PPS and dyadic- level PPS. The model is very concise but it covers almost all the elements of previous PPS measurement models. After giving a theoretical definition of PPS and the three-dimensional model, we applied it to empirically test the theoretical relationship between the process of perceived psychological safety and individual employee CSE, further validating the PPS measurement scale. The empirical analyses showed that this measure scale we developed has good internal consistency and reliability, and it has good content, convergent and discriminant validity. Although there is a growing body of research focusing on the PPS, this paper is the first to design and develop a relatively comprehensive, psychometrically sound, operationalised and validated scale for the PPS.

### Theoretical and managerial insights

The findings of this paper are also instructive for future research. Theoretically the contribution of this paper is to deepen the understanding of PPS and to some extent to develop the theory of PPS. Based on an analysis of the nature of PPS and its connotations, the paper identifies three key dimensions of PPS from both theoretical and empirical perspectives: organisational- level PPS, team- level PPS and dyadic- level PPS. Although some of these ideas reflect the perspectives of previous researchers and interviewees, the most important value of this paper is the organic integration of the different perspectives into a three-dimensional model that provides a set of empirically tested, verifiable, reliable and valid scales. The relevant findings are very encouraging and lay a solid foundation for further relevant theoretical and empirical research around PPS in the future.

Understanding PPS and how to effectively measure it is exceptionally important for business managers and their related practitioners. Using the validated scale provided in this study, they can determine how well they are doing in managing the close relationship with their employees. By applying this scale on a regular basis, management practitioners can dynamically track the trajectory and trends of PPS at the organisational- level, PPS at the team- level and PPS at the dyadic- level over time. At the same time, the scale provided in this paper can be used as an effective diagnostic tool to identify specific areas for improvement and to highlight activities that need to be focussed on in the practice of employee relations management. In addition, they can also identify employees with high PPS and design targeted strategies to maintain high PPS levels, or identify employees with low PPS who are important to the long-term development of the organisation and design effective strategies to improve PPS levels. It can be said that the collection and use of the above three aspects of PPS information has a significant impact on the success or failure of employee relationship management.

### Limitations and future research directions

Although this paper has drawn insightful conclusions about the PPS, it is also important to recognise the shortcomings of this paper. For example, since all the data involved in the study came from the same respondents, Common Method Variance (CMV) may be unavoidable, despite the fact that we used methods such as anonymous completion and disrupting and randomising the question entries in the scale development process. For this reason, we conducted a one-way test of the Harman [65], which showed that the issue of homologous variance was not a significant problem in this study. Nonetheless, it is important for future studies to consider a combination of measures based on field observations or trials, etc. Meanwhile, although the scales regarding organisation- level PPS, team- level PPS and dyadic- level PPS look encouraging, given that PPS is a very complex construct, there is still a need to further improve the quality of the scales in future research. In addition, the main objective of this paper is to develop a reliable and valid PPS scale and validate it, which can be used as a basis for future research to further explore the antecedents of the PPS and the CSE outcomes of individual employee creativity. Moreover, future research can also further analyse the role of mediating and moderating variables in the relationship between PPS and CSE outcomes of individual employee creativity.

## Electronic supplementary material

Below is the link to the electronic supplementary material.

Supplementary Material 1

## Data Availability

The datasets used and/or analysed during the current study available from the corresponding author on reasonable request.

## Abbreviations

ICC: Intra-class correlation coefficients
PPS: Perception of psychological safety
TPS: Team psychological safety
EFA: Exploratory Factor Analysis
CFA: Confirmatory Factor Analysis
CSE: Creative self- efficacy
PPS- Scale: Perception of Psychological Safety Scale
TPS- Scale: Team psychological safety Scale
