# Supplementary material for: The employee’s perception of psychological safety: construct dimensions, scale development and validation
Source: BMC Psychol. 2024 Dec 21;12:770. doi: 10.1186/s40359-024-02295-3 (PMC11662502; doi:10.1186/s40359-024-02295-3)
Supplement: Supplementary file 1 — Supplementary Material 1 [file 40359_2024_2295_MOESM1_ESM.docx]

**Appendix 1 - Questionnaire**

**The Study from Scale Development of the Employee’s Perception of Psychological Safety and Validation**

**Dear Respondent**

Thank you for participating in this thesis survey. The data collected by this questionnaire will be used for research purposes only. Our statistical analysis is based on the combined results of all questionnaires and no personal data will be analysed or stored. Therefore, please answer all questions based on your own personal experience and true feelings. This questionnaire consists of two parts: 1) the background of the respondents and 2) the evaluation of the respondents. It will take approximately 20 minutes to complete the questionnaire. Thank you very much for your support and help with this academic research.

Company name : Date of questionnaire:

# **Part I: Background information on the respondent**

# Please provide the following background information to help us better understand the study result.

# Gender: Male ; Female .

# Age .

# Your occupation:

# General staff members.

# Supervisors.

# Managers.

# General managers or chairpersons

# Highest education:

# Graduate degree or above.

# Bachelor's degree.

# college diploma.

# High school education

**Part II: Question and Answer Section**

Please circle 'O' and select a number to indicate how much you agree with each statement below (1 for completely inconsistent, 5 for completely consistent). There are no objective answers or scoring criteria. Please judge and estimate based on your experience, feelings and observations. Please answer all questions.

| Dimension | Item | | | | | |
| --- | --- | --- | --- | --- | --- | --- |
| Perceptions of psychological safety at organizational level | POPS1 In the workplace, we fully understand our work goals or roles. | 1 | 2 | 3 | 4 | 5 |
|  | POPS2 In the workplace, we can freely express our views without taking risks. | 1 | 2 | 3 | 4 | 5 |
|  | POPS3 In the workplace, we always receives organizational support and assistance when we encounter difficulties. | 1 | 2 | 3 | 4 | 5 |
|  | POPS4 In the workplace, we can use different methods to solve work problems | 1 | 2 | 3 | 4 | 5 |
|  | POPS5 In the workplace, we can freely express our opinions when communicating about specific work tasks. | 1 | 2 | 3 | 4 | 5 |
| Perceptions of psychological safety at team level | PTPS1 In the workplace, our team members become familiar with each other through social discussions. | 1 | 2 | 3 | 4 | 5 |
|  | PTPS2 In the workplace, our team leaders always provide me with clear and consistent work guidance plans. | 1 | 2 | 3 | 4 | 5 |
|  | PTPS3 In the workplace, our team leaders always refer to the opinions of their members when making decisions. | 1 | 2 | 3 | 4 | 5 |
|  | PTPS4 In the workplace, our team leaders always share the joy of success or the frustration of failure with us. | 1 | 2 | 3 | 4 | 5 |
| Perceptions of psychological safety at dyadic level | PIPS1 In the workplace, I can be my true self. | 1 | 2 | 3 | 4 | 5 |
|  | PIPS2 In the workplace, my team cares about my health. | 1 | 2 | 3 | 4 | 5 |
|  | PIPS3 In the workplace, my manager considers my perspective when making decisions. | 1 | 2 | 3 | 4 | 5 |
|  | PIPS4 In the workplace, the work I do has a significant impact on my team's performance. | 1 | 2 | 3 | 4 | 5 |
|  | PIPS5 In the workplace, I can choose the work style that suits me. | 1 | 2 | 3 | 4 | 5 |
|  | PIPS6 In the workplace, I can always perceive a clear development of the company. | 1 | 2 | 3 | 4 | 5 |
